# Bortezomib, a Proteasome Inhibitor, Attenuates Angiotensin II-Induced Hypertension and Aortic Remodeling in Rats

**DOI:** 10.1371/journal.pone.0078564

**Published:** 2013-10-30

**Authors:** Shuai Li, Xuejun Wang, Yifan Li, Curtis K. Kost, Douglas S. Martin

**Affiliations:** Basic Biomedical Sciences, Sanford School of Medicine, University of South Dakota, Vermillion, South Dakota, United States of America; Max-Delbrück Center for Molecular Medicine (MDC), Germany

## Abstract

**Background:**

Hypertension is a highly prevalent disorder and a major risk factor for cardiovascular diseases. Hypertensive vascular remodeling is the pathological mal-adaption of blood vessels to the hypertensive condition that contributes to further development of high blood pressure and end-organ damage. Hypertensive remodeling involves, at least in part, changes in protein turnover. The ubiquitin proteasome system (UPS) is a major protein quality and quantity control system. This study tested the hypothesis that the proteasome inhibitor, bortezomib, would attenuate AngII-induced hypertension and its sequelae such as aortic remodeling in rats.

**Methodology/Principal Findings:**

Male Sprague Dawley rats were subjected to AngII infusion for two weeks in the absence or presence of bortezomib. Mean arterial pressure was measured in conscious rats. Aortic tissue was collected for estimation of wall area, collagen deposition and expression of tissue inhibitors of matrix metalloproteases (TIMP), Ki67 (a marker of proliferation), reactive oxygen species (ROS) and VCAM-1 (a marker of inflammation). AngII infusion increased arterial pressure significantly (160±4 mmHg vs. vehicle treatment 133±2 mmHg). This hypertensive response was attenuated by bortezomib (138±5 mmHg). AngII hypertension was associated with significant increases in aortic wall to lumen ratio (∼29%), collagen deposition (∼14%) and expression of TIMP1 and TIMP2. AngII also increased MMP2 activity, proteasomal chymotrypsin-like activity, Ki67 staining, ROS generation and VCAM-1 immunoreactivity. Co-treatment of AngII-infused rats with bortezomib attenuated these AngII-induced responses.

**Conclusions:**

Collectively, these data support the idea that proteasome activity contributes to AngII-induced hypertension and hypertensive aortic vascular remodeling at least in part by modulating TIMP1/2 and MMP2 function. Preliminary observations are consistent with a role for ROS, inflammatory and proliferative mechanisms in this effect. Further understanding of the mechanisms by which the proteasome is involved in hypertension and vascular structural remodeling may reveal novel targets for pharmacological treatment of hypertension, hypertensive remodeling or both.

## Introduction

Hypertension is a major health care issue affecting 30% of adults in the United States [1]. Hypertension is also a major risk factor for coronary artery disease, stroke, heart failure and renal failure [1], [2]. Under hypertensive conditions, structural remodeling in blood vessels participates critically in the development and maintenance of hypertension and end-organ damage [3]–[5]. Hypertension triggers various types of arterial structural remodeling including hypertrophic remodeling [3]–[6]. Remodeling eventually leads to increased wall to lumen diameter ratio, which is a major finding in established hypertension [7]. Increased wall to lumen diameter ratio may contribute to both enhanced vascular reactivity and vascular stiffness, two cardinal features of hypertension-associated vascular pathology that are thought to contribute to the progression of this disease [4], [7]. These structural adaptations may involve reorganization of both intracellular (e.g. vascular smooth muscle cell hyperplasia/hypertrophy) [8], [9] and extracellular (e.g. changes in extracellular matrix) protein content [10]. Accumulating evidence suggests that an imbalance in matrix metalloproteases (MMPs) and their cognate inhibitors (tissue inhibitors of matrix metalloproteases; TIMPs) contribute importantly to vascular sequelae of hypertension [11], [12]. Indeed, manipulation of TIMP function has been proposed as a mechanism to attenuate hypertension induced vascular damage [12].

Recent evidence suggests that protein quality and quantity control systems play key roles in human health [13]. The ubiquitin proteasome system (UPS) is a major protein quality and quantity control system. The UPS has been implicated in cardiac remodeling associated with heart failure [13]–[15] and vascular remodeling associated with atherosclerosis [16] and potentially other cardiovascular diseases [17]. Indeed, recently reviewed evidence suggests that the vascular UPS system may play a multifactorial and powerful role in vascular smooth muscle control [18]. Hypertension-induced structural changes in blood vessels involve reorganization of both cellular and extracellular proteins, so it seems logical that the UPS should be involved with these pathologic changes. Nevertheless, this idea has received only limited research attention. Early work suggested that proteasome inhibition attenuated hypertension development and aortic remodeling in DOCA salt hypertension [19], [20]. Similarly, proteasome inhibition improved endothelial function and reduced blood pressure in AngII-infused mice [21]. Treatment with proteasome inhibitors was also reported to reduce vascular superoxide generation and inflammation in Dahl salt sensitive hypertension [22], but did not reduce blood pressure in this model. Hypertension was not reduced by proteasome inhibition in spontaneously hypertensive rats [23]. In humans, the use of proteasome inhibitors for the treatment of cancer was associated with either increases or decreases in systemic blood pressure [24]. Thus, proteasome function may be involved in blood pressure control and, potentially blood pressure dysregulation. This study tested the general hypothesis that the proteasome inhibitor, bortezomib, would attenuate AngII-induced hypertension and its sequelae such as aortic remodeling in rats.

## Materials and Methods

### *In Vivo* Experiments

These studies used male Sprague Dawley rats. All procedures involving these animals were reviewed and approved by the institutional animal care and use committee of the University of South Dakota (Protocol # 75-08-10-13D) and conform to the Guide for the Care and Use of Laboratory Animals. At the age of 14 weeks, the male Sprague Dawley rats (Harlan) were anesthetized with isoflurane (2–3% in oxygen) and osmotic pumps (Alzet, Model 2ML2were implanted subcutaneously. AngII in 0.9% saline (Sigma-Aldrich, MO) or saline were administrated by osmotic pumps for 14 days. Bortezomib (**Bort**) (LC Laboratories, MA) was dissolved in 20% cyclodextrin (Sigma-Aldrich, MO) and injected intraperitoneally (I.P.). The rats were divided into four treatment groups (N = 5 each). Group 1 was treated with vehicle (**Veh**; 0.9% saline+20% cyclodextrin). Group 2 was treated with AngII (**AngII**; AngII 200 ng/kg/min and 20% cyclodextrin). Group 3 was treated with bortezomib (**Bort**; 200 µg/kg, 3 times per week, I.P. and received 0.9% saline subcutaneously). Group 4 was treated with a combination of AngII infusion and I.P. bortezomib (**AngII/Bort**). Body weight was measured prior to the start and at the end of treatments. At day 12 of treatment, rats were anesthetized and carotid artery catheters were implanted. Buprenorphine (0.03 mg/kg) was provided for postoperative analgesia. After 24 hours of recovery, the arterial catheters were connected to a data acquisition system (Biopac Systems, CA) and blood pressures were recorded during daylight hours when the rats were conscious and unrestrained. Average mean arterial blood pressure (MAP) of 10 time points equally distributed over three hours was used for statistical analysis. Rats were euthanized (pentobarbital 150 mg/kg) at day 14. The last dose of bortezomib was delivered on the morning of day 14, approximately 8 hours before tissue collection. The thoracic aorta (from aortic arch to diaphragm) and tibialis anterior skeletal muscle samples were collected, and snap frozen in dry ice or fixed in 4% paraformaldehyde.

### Proteasome activity assay

Frozen tibialis anterior skeletal muscle was homogenized in Hepes buffer (50 mmol/L). Following centrifugation, the supernatant was collected and assayed for protein concentration. 30 µg of sample protein was added with fluorogenic proteasome substrate III (Suc-LLVY-AMC, chymotrypsin-like, 18 µM, Calbiochem, EMD biosciences, CA) with or without the proteasome inhibitor MG132 as a total inhibition control (20 uM, Sigma-Aldrich, MO) and incubated at 37°C for 30 minutes. The reaction was then quenched by adding ice-cold ethanol followed by water. The samples were read at excitation wavelength of 350 nm and emission wavelength of 435 nm. The chymotrypsin-like proteasome activity was calculated as the generated fluorescence strength difference between samples with and without MG132.

### Masson's trichrome staining

Paraformaldehyde fixed aorta samples were sectioned (10 um) at minus 20°C with a cryostat (Leica, MN) and then mounted on slides. Slides underwent a series of incubations in Masson's trichrome staining solutions using standard protocols (Sigma-Aldrich, MO). The slides were then dehydrated with ethanol and rinsed with Citrus Solvent. Pictures of stained aorta sample sections were taken with light microscope (5X, 100X). At 5× magnification, the medial cross sectional area and the lumen area of the aorta were measured with ImageJ software. The medial cross sectional area to lumen area ratio was used as an index of vascular structural remodeling. At 100× magnification, using specific Masson's trichrome filter, the blue color density within vascular smooth muscle wall area was quantified with ImageJ software and used as an index of ECM deposition.

### Western blot analysis

Aorta samples were homogenized in RIPA buffer with protease and phosphatase inhibitor cocktail (Thermo Scientific, IL) using Bullet Blender homogenizer (Nextadvance, NY). After centrifugation, clear supernatant was collected. The protein concentration was evaluated by BCA protein assay (Thermo Scientific, IL). 30 ug of sample protein was mixed with 4× Protein Loading Buffer (Li-cor, NE) and subjected to SDS PAGE gel electrophoresis and transferred to polyvinylidene difluoride (PVDF) membrane (Bio-Rad, CA). The PVDF membranes were blocked in Aqua Block blocking buffer (EastCoast Bio, ME) overnight. Primary antibodies against proteins of interest were diluted in Aqua Block and incubated with the membranes overnight at 4°C (TIMP1, 1∶500, AbD Serotec, UK; TIMP2, 1∶500, Novus Biologicals, CO; beta-Actin, 1∶2000, Li-cor, NE). Fluorescent-labeled secondary antibodies were incubated with the membranes for three hours at 4°C (IRDye 680LT Conjugated Goat polyclonal Anti-Mouse IgG, 1∶5000, IRDye 800LT Conjugated Goat polyclonal Anti-Rabbit IgG, 1∶5000, Li-cor, NE). The membranes were scanned with the Odyssey Infrared Imager (LiCor Biosciences, NE). Densitometric quantification of the bands was performed using Image Studio software (LiCor Biosciences, NE). The relative amount of targeted protein was calculated as a percentage of actin loading control.

### Gelatin zymography

Aorta samples were homogenized in RIPA buffer without protease and phosphatase inhibitors and handled as described above for Western blot. 30 ug of sample protein was mixed with 4× Protein Loading Buffer (Li-cor, NE) and loaded in each well of the SDS page gel with gelatin (1.0 mg/ml) and subjected to electrophoresis. MMP positive control (Sigma-Aldrich, MO) was added. After electrophoresis, the gelatin gel was washed twice with 2.5% Triton X-100 in water for 30 minutes. Subsequently the gel was incubated with zymography incubation solution (10 mM CaCl_2_, 50 mM Tris-Acetic Acid, pH 7.5) overnight at 37°C. The gel was stained with 0.25% Comassie Blue R-250 for 1 hour and de-stained with 10% Acetic Acid, 10% Methanol over 1 hour to develop the bands. A VersaDoc gel image system (Bio-Rad, CA) was used to capture the image. Brightness of the MMP2 bands was evaluated with ImageJ software following a suggested protocol from NIH (http://rsb.info.nih.gov/ij/docs/menus/analyze.html#gels).

### Dihydroethidium (DHE) Staining

Reaction of DHE with superoxide generates ethidium that fluoresces red. Red fluorescence was used to evaluate in situ superoxide level. Fresh frozen descending thoracic aorta samples were sectioned (30 um) at minus 20°C, mounted on slides and treated with PBS at 37°C for 30 minutes. The sections were then treated with DHE (8 uM) and transferred into a humidified dark chamber for 30 minutes at 37°C. The slides were rinsed twice with PBS (5 minutes each time) in a dark chamber. Pictures were taken immediately under a fluorescent microscope. The vascular smooth muscle wall area size and the red fluorescent intensity in this area of the aorta were measured with ImageJ software (NIH). The red fluorescence intensity level was normalized with the aortic vascular smooth muscle cell wall area size to represent the in situ superoxide level.

### Immunohistochemistry staining

Paraformaldehyde fixed descending thoracic aorta samples were sectioned (5 um) at minus 20°C, mounted on slides and incubated with antigen retrieval solution (10 mM citrate, 0.05% Tween 20) in a steamer for 20 minutes. After rinsing with PBS, the sections were incubated with 0.075% hydrogen peroxide for 5 minutes. After 2 more rinses with PBS, the slides were incubated in 0.25% Triton X-100 in PBS at 4°C in humidified chamber for 48 hours, with or without primary anti Ki67 (Millipore, CA) or vascular cell adhesion molecule 1 (VCAM-1) (AbD serotec, NC) antibodies at 1∶1000 dilution. After rinsing in PBS the sections were incubated with biotinylated secondary antibodies (Vector, CA) at 1∶1000 at 4°C for 4 hours. Following PBS rinses, the sections were incubated with ABC reagent (Vector, CA) for 1 hour at room temperature, then the sections were stained with 3.3′-diaminobenzidine containing 0.01% hydrogen peroxide for 20 minutes. Pictures were taken under light microscope and the brown reaction product analyzed with ImageJ software (NIH).

### Statistics

Statistical data analysis was performed with Graph Pad Prism 4. Analysis of variance (ANOVA) was followed by Student Newman-Keuls post hoc test when appropriate. Statistical significance was identified at p<0.05. Data are expressed as the mean± SEM.

## Results

### Bortezomib treatment attenuated AngII-induced hypertension

**Figure 1:**
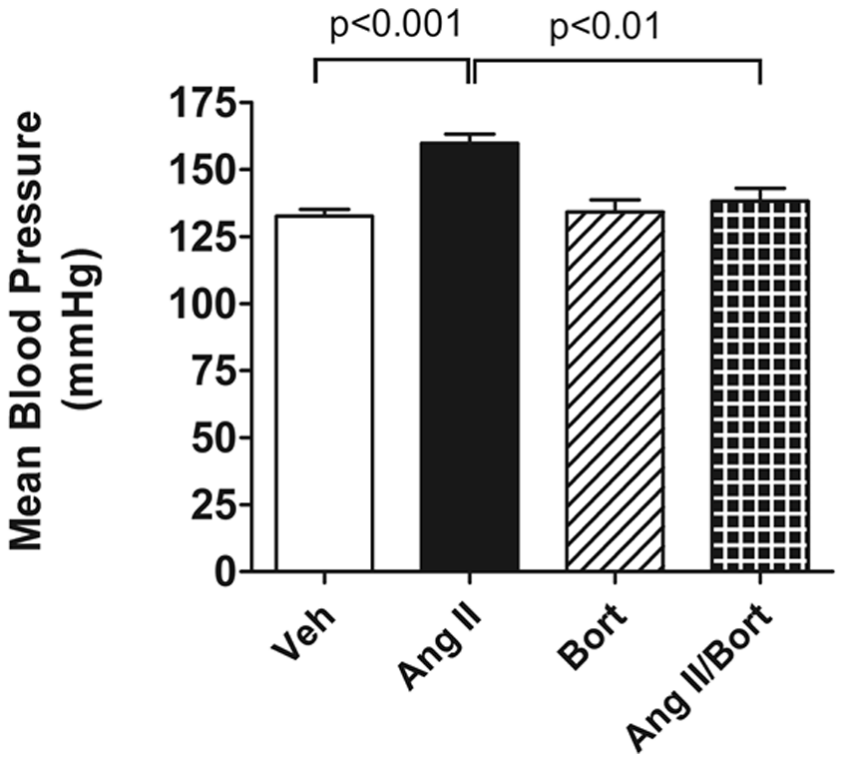
Effect of Bortezomib on AngII-induced Hypertension. This figure shows mean arterial pressure in conscious rats 13 days after vehicle saline and vehicle cyclodextrin treatment (Veh), AngII continuous infusion (AngII), bortezomib treatment (Bort) or combined AngII/Bortezomib treatment (AngII/Bort). N = 5 in each group. Overall ANOVA p = 0.0005. Post hoc analysis (Newman Keuls) for Veh versus Ang II and AngII versus AngII+Bort comparisons shown in the figure.

AngII treated rats showed significantly greater MAP (160±4 mmHg) compared to Veh treated rats (133±2 mmHg). Bortezomib treatment alone did not change MAP compared to vehicle (134±5 mmHg). In contrast, co-treatment of the AngII-infused rats with the proteasome inhibitor resulted in a MAP (138±5 mmHg) that was lower than that observed in the AngII treated rats, but not significantly different from that in the Veh or Bort treatment groups (Figure 1). Thus, bortezomib treatment attenuated AngII-induced hypertension in SD rats. Body weight was not significantly affected by the treatments in any of the four groups (Table 1).

**Table 1:** Body Weights

Body Weight	Veh	Ang II	Bort	AngII/Bort
**Baseline (g)**	**414±11**	**403±10**	**399±9**	**384±20**
**Treatment (g)**	**416±7**	**404±15**	**405±8**	**375±21**
**Treatment/Baseline Ratio**	**1.01±0.01**	**1.00±0.02**	**1.02±0.02**	**0.98±0.02**

This table shows the body weights at baseline before the start of treatments and at the conclusion of the treatment period. Veh: vehicle, Ang II: angiotensin, Bort: bortezomib, AngII/Bort: concurrent treatment with angiotensin and bortezomib. There were no statistically significant differences amongst the treatment groups.

**Figure 2:**
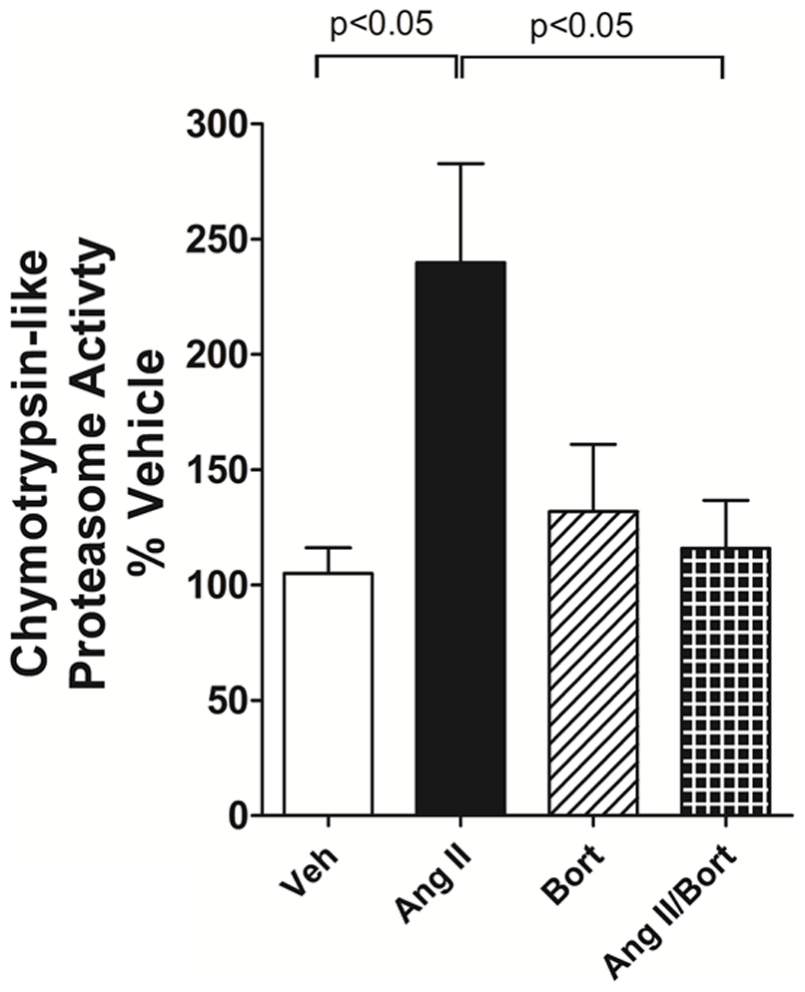
Chymotrypsin-like proteasome activity in rat tibialis anterior skeletal muscle. Rats were sacrificed and tibialis anterior skeletal muscle samples were collected 14 days after vehicle saline and vehicle cyclodextrin treatment (Veh), AngII continuous infusion (AngII), bortezomib treatment (Bort) or combined AngII/Bortezomib treatment (AngII/Bort). N = 5. Overall ANOVA p = 0.015. Post hoc analysis (Newman Keuls) for Veh versus Ang II and AngII versus AngII+Bort comparisons shown in the figure

The effectiveness of bortezomib treatment on proteasome function was assessed by measuring skeletal muscle chymotrypsin-like activity. AngII treatment activated chymotrypsin-like UPS activity in tibialis anterior skeletal muscle compared to Veh treated rats. Treatment with bortezomib alone did not elicit a major change in proteasome activity, suggesting that basal proteasome function was not impaired. However, co-treatment of the AngII-infused rats with bortezomib significantly attenuated AngII-induced activation of chymotrypsin-like proteasome activity (Figure 2).

### Bortezomib treatment attenuated AngII-induced aorta vascular remodeling

**Figure 3:**
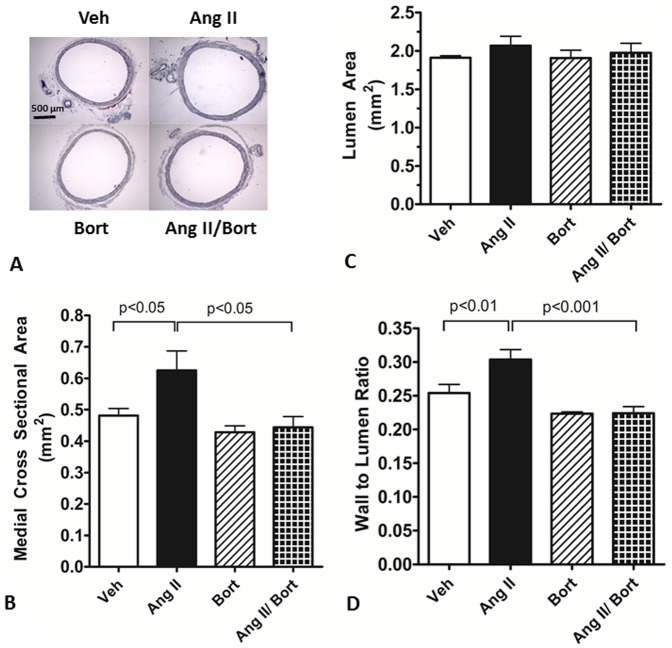
Effect of Bortezomib on AngII-induced aortic remodeling. Figure 3A shows photos of Masson's trichrome staining of aortas (5× magnification). Figure 3B shows the medial cross sectional area. Figure 3C shows the aorta inner lumen area. Figure 3D shows the aorta medial cross sectional area to inner lumen area ratio. Vehicle saline and vehicle cyclodextrin treatment (Veh), AngII continuous infusion (AngII), bortezomib treatment (Bort) or combined AngII and bortezomib treatment (AngII/Bort). N = 5. Overall ANOVA Fig. 3B p = 0.01, Fig. 3C p = 0.66, Fig. 3D p = 0.0003. Post hoc analysis (Newman Keuls) for Veh versus Ang II and AngII versus AngII+Bort comparisons shown in each figure

The wall-to-lumen ratio was used as an index of aortic vascular remodeling (Figure 3). Ang II-induced aortic remodeling appeared to involve increased aorta medial cross sectional area (Figure 3B). There was no significant change in inner lumen area (Figure 3C) amongst the four treatment groups. However, AngII treatment significantly increased (∼20%) aorta wall-to-lumen ratio, compared to Veh treated rats (Figure 3 D). Treatment with bortezomib alone did not change the wall-to-lumen ratio. Conversely, wall to lumen ratio was decreased significantly in the AngII/Bort treated rats compared to the AngII treated rats. Thus, bortezomib treatment attenuated AngII-induced aortic vascular remodeling.

### Effect of bortezomib on extracellular matrix in aorta

**Figure 4:**
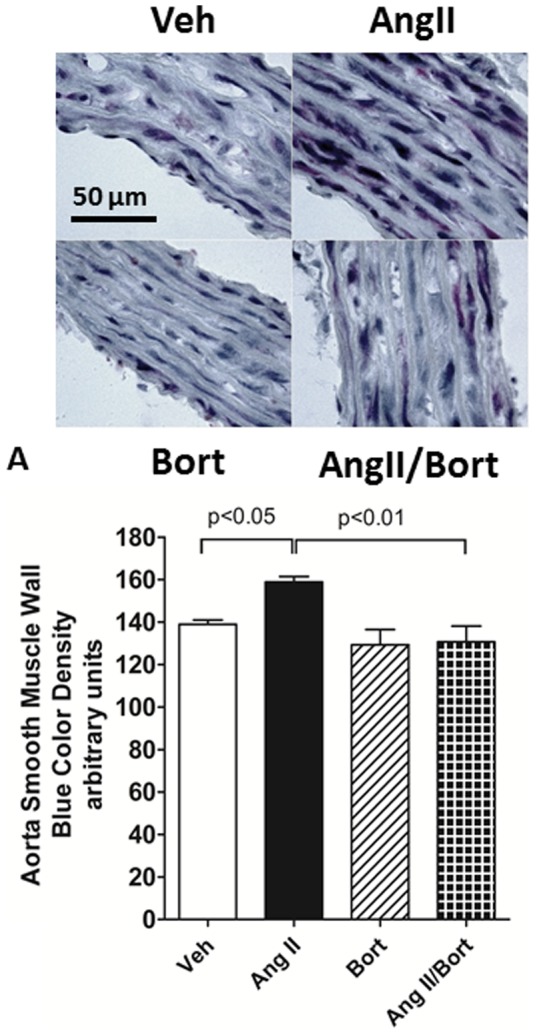
Effect of Bortezomib on Collagen Deposition in AngII hypertension. Figure 4A shows photos of Masson's trichrome staining of aortas (100X). Figure 4B represents the density of collagen staining in aortic smooth muscle wall. Vehicle saline and vehicle cyclodextrin treatment (Veh), AngII continuous infusion (AngII), bortezomib treatment (Bort) or combined AngII and bortezomib treatment (AngII/Bort). N = 4. Overall ANOVA p = 0.003. Post hoc analysis (Newman Keuls) for Veh versus Ang II and AngII versus AngII+Bort comparisons shown in the figure.

Masson's trichrome staining was used to estimate overall collagen deposition as indicated by the density of blue staining (Figure 4A). Staining density was increased in AngII treated rat aorta compared to aorta obtained from Veh treated rats (Figure 4B), consistent with increased aortic collagen deposition in AngII treated rats. Aorta harvested from rats treated with bortezomib alone showed staining density comparable to vehicle treated rats. Staining density in aorta from AngII/Bort treated rats was markedly reduced compared to that observed in the AngII treated rats (Figure 4B). Thus, these data suggest that bortezomib treatment attenuated AngII-induced collagen deposition.

**Figure 5:**
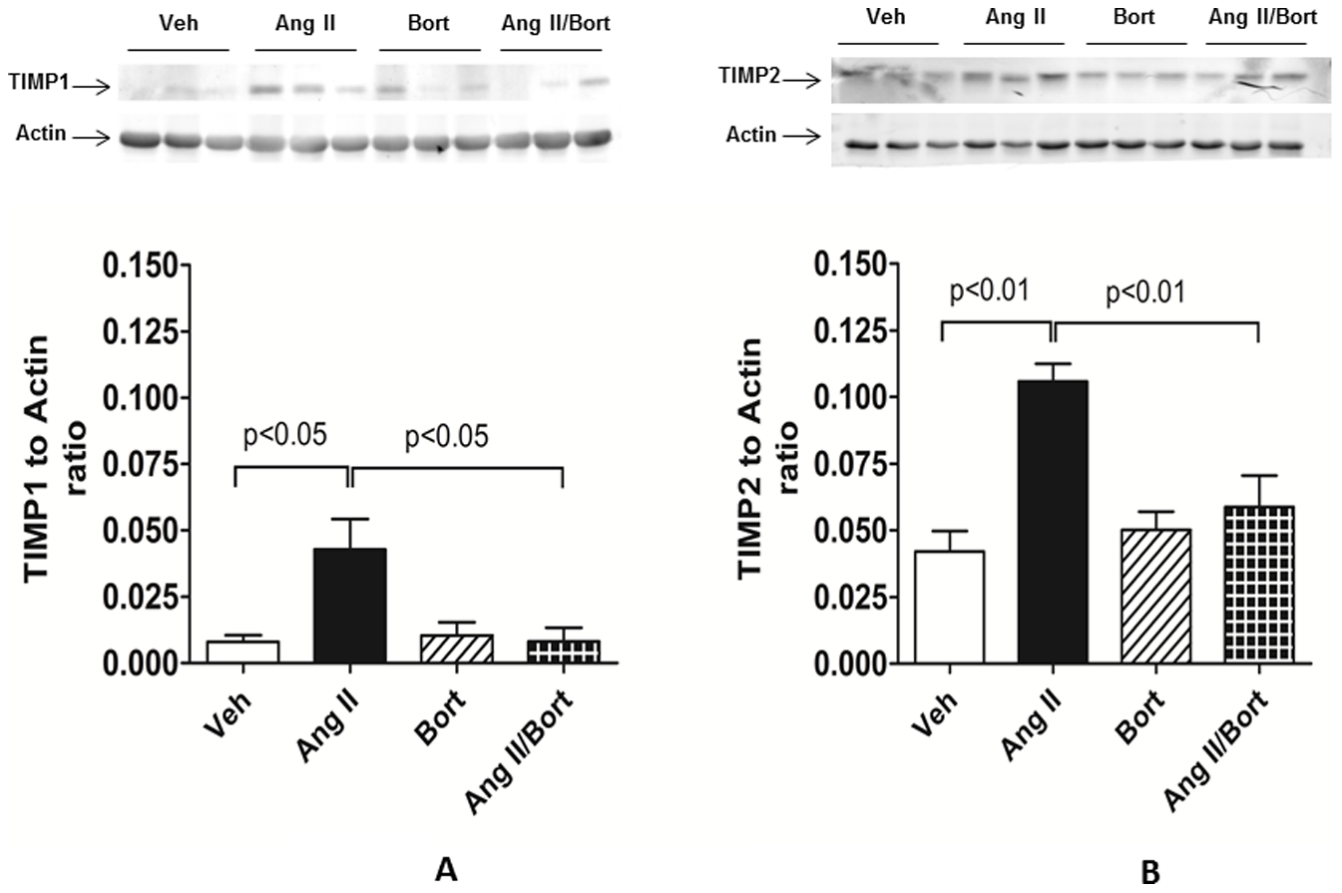
Effect of Bortezomib on AngII-induced Hypertensive Changes in TIMP Expression. For figure 5A, the upper panel is the western blot result of TIMP1 in aorta and the lower panel is a summary of densitometric values. For figure 5B, the upper panel is the western blot result of TIMP2 in aorta with the lower panel as the densitometry. Vehicle saline and vehicle cyclodextrin treatment (Veh), AngII continuous infusion (AngII), bortezomib treatment (Bort), AngII and bortezomib treatment (AngII/Bort). N = 3. Overall ANOVA Fig. 5A p = 0.02, Fig. 5B p = 0.003. Post hoc analysis (Newman Keuls) for Veh versus Ang II and AngII versus AngII+Bort comparisons shown in each figure.

**Figure 6:**
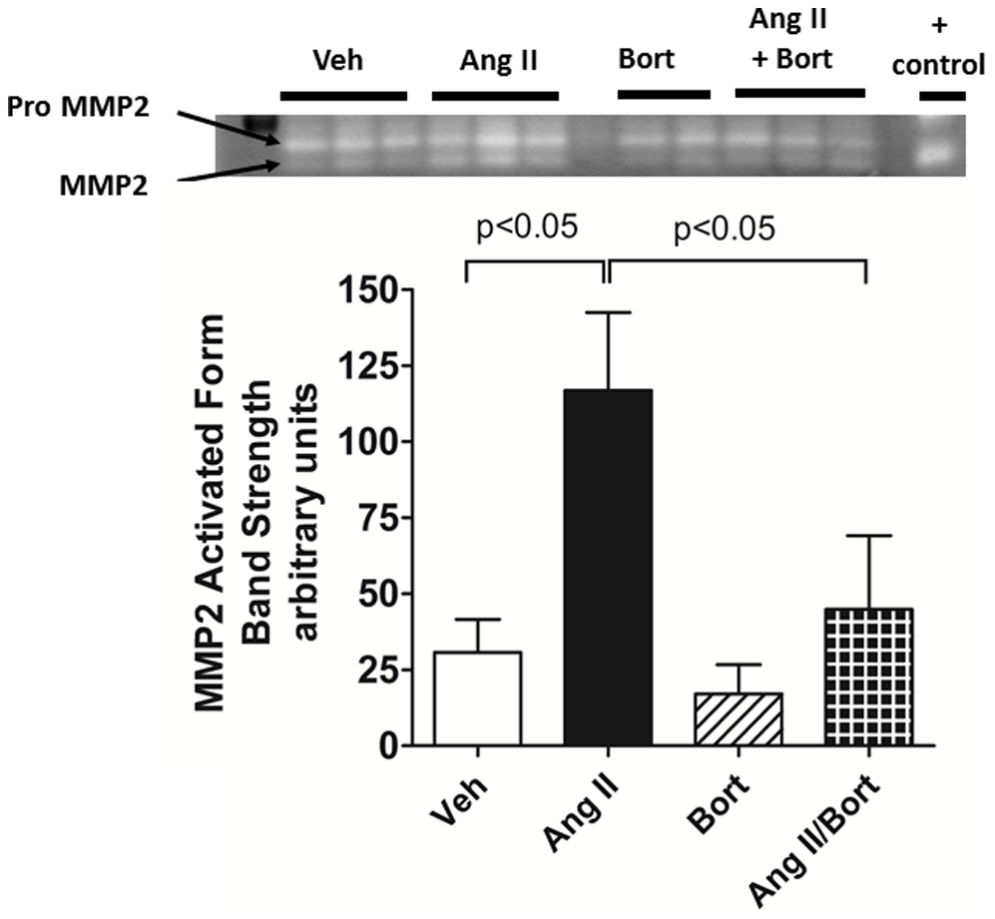
Effect of Bortezomib on AngII-induced Hypertensive Changes in MMP2 Activity. The image of MMP2 zymography gel is shown in the upper panel. The lower panel shows summary data for the brightness of the bands representing the activated forms of MMP2. Vehicle saline and vehicle cyclodextrin treatment (Veh), AngII continuous infusion (AngII), bortezomib treatment (Bort), AngII and bortezomib treatment (AngII/Bort). N = 3. Overall ANOVA p = 0.02. Post hoc analysis (Newman Keuls) for Veh versus Ang II and AngII versus AngII+Bort comparisons shown in the figure.

Matrix metalloproteases (MMPs) along with their cognate tissue inhibitors of matrix metalloproteases (TIMP) play an important role in the control of extracellular matrix collagen turnover. AngII-treatment was associated with an increase in protein expression of both TIMP1 (Figure 5A) and TIMP2 (Figure 5B) compared to the Veh treatment group. Bortezomib alone did not change TIMP1 or TIMP2 protein amounts, but combined treatment with bortezomib and AngII resulted in aortic TIMP1 and TIMP2 protein levels that were markedly reduced compared to the AngII treated rats. We used zymography to assess potential changes in MMP activity. Somewhat unexpectedly we observed that AngII treatment increased MMP2 activity (Figure 6). Treatment with bortezomib alone did not affect MMP2 activity compared to vehicle treated rats. However, the MMP2 activation observed in aorta from AngII-treated rats was reversed when rats were co-treated with AngII and bortezomib. MMP2 protein level as assessed by western blot was not significantly affected in any of the treatment groups (data not shown).

### Effect of bortezomib on an index of cellular proliferation in aorta

**Figure 7:**
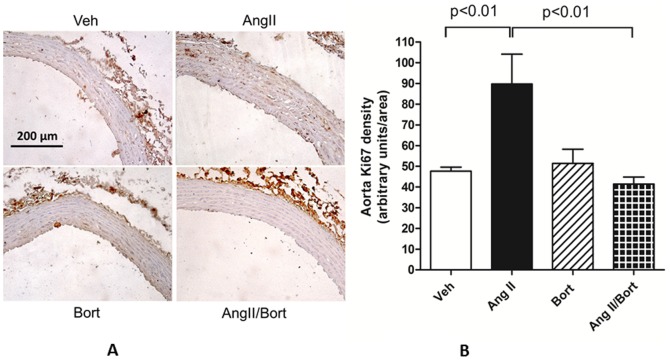
Effect of bortezomib on an index of cellular proliferation in aorta. Images of Ki67 staining in the aorta from each group are illustrated on the left. The right side shows summary data for each group. Vehicle saline and vehicle cyclodextrin treatment (Veh), AngII continuous infusion (AngII), bortezomib treatment (Bort), AngII and bortezomib treatment (AngII/Bort). N = 3. Overall ANOVA p = 0.005. Post hoc analysis (Newman Keuls) for Veh versus Ang II and AngII versus AngII+Bort comparisons shown in the figure.

Ki67 immunoreactivity was used as an index of cellular proliferation (Figure 7A). Ki67 immunoreactivity was increased approximately 2 fold in aorta harvested from AngII hypertensive rats compared to those obtained from vehicle treated rats. In rats treated with both AngII and bortezomib, Ki67 immunoreactivity averaged approximately 45% of that observed in the AngII treated rats and was slightly but not significantly reduced (∼12%) compared to vehicle treated rats (Figure 7B).

### Effect of bortezomib on aortic ROS and VCAM expression

**Figure 8:**
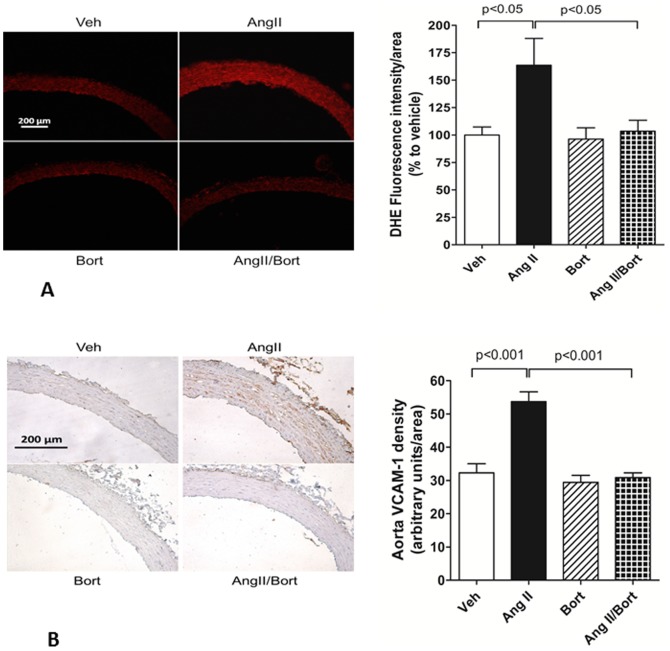
Effect of bortezomib on aortic ROS and VCAM staining. The top panel A shows images of dihydroethidium (DHE) staining (red) in aorta on the left and summary data for each group on the right. The lower panel side B show individual images for VCAM-1 staining (brown reaction product) in aorta on the left and summary data for VCAM-1 staining on the right. Vehicle saline and vehicle cyclodextrin treatment (Veh), AngII continuous infusion (AngII), bortezomib treatment (Bort), AngII and bortezomib treatment (AngII/Bort). N = 3. Overall ANOVA Fig. 8A p = 0.02, Fig. 8B p<0.0001. Post hoc analysis (Newman Keuls) for Veh versus Ang II and AngII versus AngII+Bort comparisons shown in each figure.

Aortic ROS generation was estimated by dihydroethidium staining. DHE staining was increased approximately 1.7 fold in aortae harvested from AngII hypertensive rats compared to vehicle treated rats (Figure 8A). Bortezomib treatment alone did not alter DHE staining intensity. On the other hand, DHE staining was markedly attenuated in aortae harvested from rats treated concurrently with AngII and bortezomib compared to the AngII treated rats. VCAM-1 staining was used as an index of inflammation (Figure 8B). VCAM-1 immunoreactivity was negligible in vehicle treated rats. AngII treatment increased VCAM staining significantly (∼65%). This effect was largely abrogated by concurrent treatment with bortezomib.

## Discussion

This study tested the general hypothesis that the proteasome inhibitor, bortezomib, would attenuate AngII-induced hypertension and its sequelae such as aortic remodeling in rats. The data showed that co-treatment with bortezomib attenuated AngII-induced hypertension and aortic vascular remodeling. Bortezomib also prevented the increase in aortic collagen deposition associated with AngII hypertension. AngII infusion increased TIMP1 and TIMP2 protein levels in aorta concurrent with activation of MMP2. AngII treatment also increased indices of aortic cell prolilferation, superoxide generation and inflammation. These responses were all attenuated when bortezomib was co-administered with AngII. Collectively these data suggest that, bortezomib treatment attenuated many of the events downstream of AngII stimulation that are associated with hypertension and hypertensive aortic remodeling.

We used an induced model of hypertension, the AngII infusion model that exhibits a gradual increase in MAP that stabilizes at hypertensive levels in about 72 hours [25], [26]. At day 12 of AngII infusion we observed an increase in MAP of approximately 30 mmHg. This value compares favorably with previous work using a similar approach [25]. We found that hypertension did not develop in AngII infused rats co-treated with the proteasome inhibitor, bortezomib. Previous work examining the effects of proteasome inhibitors on hypertension is limited. Takaoka used a DOCA salt model that exhibited an increase in systolic arterial pressure of approximately 50 mmHg. Treatment with the proteasome inhibitor, PSI (*N*-benzyloxycarbonyl-Ile-Glu (*O*-*t*-Bu)-Ala-leucinal), markedly attenuated hypertension in DOCA salt treated rats [19], [20]. Similarly, in mice AngII infusion for 14 days increased MAP by approximately 30 mmHg. Two days of concurrent proteasome inhibitor (MG132) treatment produced a significant attenuation of hypertension [21]. In contrast, a study in Dahl rats reported that bortezomib produced a modest but non-significant decrease in salt dependent hypertension [27]. Similarly, treatment of spontaneously hypertensive rats with another proteasome inhibitor, MG132 (100 µg/kg) also failed to reduce blood pressure [23]. The precise reasons for these discrepant findings are not immediately clear but may involve methodological differences. The dose of bortezomib used in the present study (200 µg/kg), a dose in the clinical range [28], was substantially higher than the doses of proteasome inhibitors used in Dahl rats or spontaneously hypertensive rats. While we cannot rule out a potential toxic effect of bortezomib at the dose we used, consistent with previous work using 200 µg/kg [28], we did not observe any overt signs of toxicity, such as weight loss. Although peripheral neuropathy was reported at this dose [28], we did not observe any overt signs consistent with this possibility. Alternatively, the different outcomes may represent differences in the fundamental mechanisms involved in these different models of hypertension. In any case the evidence currently supports the possibility that proteasome inhibition has an antihypertensive effect, at least in some forms of hypertension. Interestingly, these findings were obtained with three different inhibitors suggesting a general effect of proteasome inhibition *per se* and not the specific effects of individual drugs.

Since AngII-induced activation of chymotrypsin-like activity in skeletal muscle was previously reported (e.g. [29]), we used this as a marker of effectiveness of the bortezomib treatment. As expected we found that AngII infusion was associated with an increase in chymotrypsin-like activity [21]. This effect was largely attenuated by concurrent treatment with bortezomib. We had predicted that the bortezomib treatment alone would reduce chymotrypsin-like activity. Unexpectedly, we observed that bortezomib treatment alone did not affect basal chymotrypsin-like activity. One potential explanation for this observation is found in the work of Meiners, who reported that chronic inhibition of proteasome function triggers a compensatory upregulation of proteasome subunit expression [30]. Alternatively, subtypes of proteasomes are known to exist. These subtypes have differential sensitivities to proteasome inhibitors such as bortezomib [31]. Thus, Ang II may selectively activate a proteasome subtype that is preferentially sensitive to bortezomib. In any case, our data show that the dosing schedule used provided effective inhibition of proteasome function under AngII-stimulated conditions.

In the aorta, vascular remodeling impacts pulse pressure and end-organ damage [32], [33], an important consequence of hypertension. Vascular remodeling requires reorganization of extracellular and intracellular protein. Thus, ubiquitin-proteasome machinery involvement in hypertensive vascular remodeling is possible. However, few studies have examined this intriguing possibility. Hypertrophic remodeling involving an increase in vascular smooth muscle cross sectional area was reported in genetic [34] and induced hypertensive animal models [9], [35] and in human hypertensives [36]. We observed that chronic AngII infusion was associated with a significant increase in medial cross sectional area (∼29%) and wall to lumen ratio (∼19%). This finding is similar to an approximate 20% increase in medial cross sectional area after two weeks of AngII infusion at 250 ng/kg/min [37]. We also found that bortezomib co-treatment markedly attenuated the AngII-induced aortic hypertrophy compared to the AngII-treatment group. This outcome is consistent with previous work. In DOCA salt hypertensive rats that exhibited a 25% increase in aortic wall-to-lumen ratio, treatment with a proteasome inhibitor suppressed aortic hypertrophy to only 5% above that of vehicle treated rats [19]. Thus, the limited data available to date suggest that proteasome inhibition attenuates hypertensive aortic remodeling.

The ECM plays an important role in hypertensive vascular remodeling [38]. Accordingly we used Masson's trichrome staining to estimate collagen content as an index of ECM accumulation. We observed an increase in collagen staining in AngII-infused rats that was consistent with previous work showing that AngII infusion increased ECM deposition [26], [39] and caused significant increases in aortic collagen content in mice [40]. Thus, the current evidence is compatible with the view that AngII infusions increase collagen accumulation in the aorta. Our data implicate the proteasome in this phenomenon since bortezomib treatment effectively attenuated AngII-induced aortic collagen accumulation.

ECM turnover is governed by a dynamic balance amongst multiple factors, including MMP and their cognate inhibitors, TIMPs. We observed AngII-induced increases in TIMP1 and TIMP2 protein expression of 4 fold and 1.7 fold after chronic AngII-treatment, values that were consistent with previous reports [11], [41]. Interestingly, we found that bortezomib co-treatment suppressed AngII-induced expression of TIMP1 and TIMP2. These data support previous work showing that TIMP expression was reduced by proteasome inhibitors in other cell types such as cardiac fibroblasts in culture or in hearts of spontaneously hypertensive rats [23]. Given the increased TIMP expression in aortic tissue collected from AngII-infused rats, we predicted that reduced MMP activity might be a mechanism underlying the increased aortic collagen deposition observed in the hypertensive rats. Contrary to our prediction, we observed that MMP2 activity was increased in the aorta harvested from rats with AngII-induced hypertension. However, this finding is not without precedent. In rats, AngII-treatment increased aortic MMP2 activity [42]. Similarly, AngII infusions increased carotid artery MMP2 gelatinase activity in mice[43]. Indeed Watts reported increases in both MMP2 activity and TIMP2 protein level in DOCA salt hypertensive rats [44]. Moreover, in hypertensive patients, plasma MMP2 activity and TIMP1 protein level showed concurrent increases [45]. Thus, the precise interaction of TIMPs and MMP is complex. In any case, we observed that co-treatment with bortezomib largely abrogated the AngII-induced increase in MMP activity. These data provide support for the view that proteasome inhibition affects the changes in aortic MMP activity associated with hypertension. Our findings are consistent with work in carotid artery atherosclerosis that reported that increased proteasome activity correlated with increased MMP expression [46]. Collectively, these data support the idea that the proteasome is critically involved in controlling the balance of TIMP1/TIMP2 expression and MMP2 activity which in turn modulates aortic extracellular matrix turnover in hypertension.

The mechanisms by which proteasome inhibition mediates these effects remain to be determined. The ubiquitin proteasome system is a pleiotropic cellular mechanism that has influences on cell cycle regulation, apoptosis, transcription, protein turnover and cell signaling (e.g. reactive oxygen species, NFkB) [14], [18]. Similarly, AngII is known to elicit its effects via multiple signaling pathways [47]. Thus, there are numerous potential interactions between proteasome inhibition and AngII signaling that could account for the current observations. While beyond the scope of the present study, each of these possibilities represents viable avenues for future follow up. Attractive possibilities include oxidative, inflammatory and proliferative mechanisms. It is known that ROS play a role in mediating the vascular actions of AngII [47]. Previous work by Stangl showed that the proteasome was involved in modulating ROS accumulation in vascular smooth muscle [22]. This group reported that bortezomib treatment lowered superoxide levels in the aorta of Dahl salt sensitive hypertensive rats. In the present study we observed that AngII-induced hypertension was associated with an increase in aortic ROS that was also abrogated by treatment with bortezomib. Previous work has suggested several possibilities for this effect. Proteasome inhibition has been shown to increase antioxidative capacity by upregulating antioxidant proteins such as SOD1 and catalase [48], [49]. Alternatively, proteasome inhibition may upregulate nitric oxide synthase expression and activity as well as nitric oxide production [50], [51] which may effectively scavenge ROS. Lastly, proteasome inhibition may transcriptionally suppress NADPH oxidase expression [52]. Irrespective of the precise mechanism by which proteasome inhibition suppresses ROS, this effect may contribute to the antihypertensive effects of proteasome inhibition since ROS are a key signaling mechanism downstream of AngII.

ROS are linked with inflammatory responses. Increasing evidence suggests that T cell mediated inflammatory mechanisms contribute to hypertension and hypertensive remodeling [53], [54]. T helper cells appear to promote inflammation and hypertension [54] while regulatory T cells are proposed to act as a “brake” on hypertensive processes by suppressing vascular inflammation. [53]. In the present study we observed that AngII hypertension-induced increases in aortic VCAM-1 immunoreactivity (a marker of inflammation) were abolished by treatment with bortezomib. These data are consistent with those of Ludwig [22] who showed previously that bortezomib treatment of Dahl salt sensitive hypertensive rats reduced both ROS and VCAM-1 expression in the aorta. Thus, proteasome inhibition appears to effectively inhibit hypertension associated aortic ROS and inflammation in a model independent manner. Whether this effect is mediated through actions on T cells remains an open question. However, at present, it is not clear if the ability of proteasome inhibition to reduce inflammation underlies the anti-hypertensive action of bortezomib. Ludwig [22] did not observe a significant reduction in blood pressure despite a marked anti-inflammatory action of bortezomib. This aspect of the action of proteasome inhibition on vascular inflammation and its link to hypertension warrants further in depth study.

Lastly, vascular hypertrophy may arise in part via increases in VSMC proliferation. AngII was shown to increase the number of proliferating VSMC in the aorta [8]. Similarly, we observed that AngII hypertension was associated with an increase in aortic immunoreactive Ki67, a marker of proliferating cells. The AngII hypertension-associated increase in Ki67 was attenuated by concurrent treatment with bortezomib. Thus, interruption of AngII induced VSMC proliferation may be one mechanism by which bortezomib prevented the aortic hypertrophic remodeling observed in the present study. Alternatively, proteasome inhibition was reported to induce apoptosis in aortic vascular smooth muscle cells in culture [55]. It is conceivable therefore that proteasome inhibitor-induced apoptosis counterbalanced the increase in proliferation observed in the AngII treated aorta to prevent the increase in wall to lumen ratio recorded in this treatment group. Since we did not measure apoptosis in the present study we cannot rule out this possibility. However, it should be noted that proteasome-induced aortic vascular smooth muscle apoptosis was noted at high concentrations (500 µM) [55]. It has been proposed that the effect of proteasome inhibition on vascular smooth muscle cell apoptosis is concentration dependent, with lower concentrations actually inducing a protective effect [18], [56]. While we did not measure plasma concentrations of bortezomib in the present study, our observation that bortezomib treatment alone did not reduce wall to lumen ratio suggests that the dose of bortezomib used was not overtly toxic to vascular smooth muscle cells. Irrespective of the precise mechanism, the data obtained in the present work is consistent with the view that proteasome inhibition can, under certain conditions, reduce hypertensive hypertrophic remodeling of the aorta.

Collectively, these data suggest that bortezomib, presumably via its ability to inhibit the proteasome, exerts an inhibitory effect on multiple AngII-mediated actions that result in hypertension and hypertension associated aortic remodeling. Thus, it is tempting to speculate that proteasomal activity is required to activate an early step in the AngII signaling cascade leading to hypertension and hypertension-induced aortic remodeling.

### Perspectives

Hypertensive vascular remodeling is an adaptive response of blood vessels to normalize wall stress. However, these structural changes can contribute to exacerbation of both hypertension and its sequelae. Hypertension-induced collagen deposition can augment the stiffness of the aorta and other large arteries to increase pulse pressure. Both increased mean arterial pressure and pulse pressure are associated with increased end-organ damage and related cardiovascular diseases [57]. Growing evidence implicates the vascular UPS system as an important mechanism controlling vascular physiology and pathophysiology [18]. In the present work, concurrent treatment with bortezomib attenuated AngII-induced hypertension, cell proliferation in the aorta, aortic ROS generation and inflammation, and the associated pathological structural changes in the aorta. Thus, these experiments suggested that proteasome activity plays a critical role in this sequence of hypertension related processes. Interestingly bortezomib treatment was also reported to attenuate pulmonary artery remodeling in pulmonary hypertension [50]. Bortezomib is a reversible proteasome inhibitor which is currently approved for treatment of cancer. Its use in this setting is associated with considerable adverse effects which likely preclude the use of bortezomib per se for the treatment of hypertension. However, further understanding of the mechanisms by which the proteasome is involved in hypertension and vascular structural remodeling may reveal novel targets that may be more selective for pharmacological treatment of hypertension, hypertensive remodeling or both.

## References

[1] Roger VL, Go AS, Lloyd-Jones DM, Adams RJ, Berry JD, et al (2011) Heart disease and stroke statistics—2011 update: a report from the American Heart Association. Circulation 123: e18–e209. 21160056 10.1161/CIR.0b013e3182009701 PMC4418670

[2] Mancia G, Messerli F, Bakris G, Zhou Q, Champion A, et al (2007) Blood pressure control and improved cardiovascular outcomes in the International Verapamil SR-Trandolapril Study. Hypertension 50: 299–305. 17606861 10.1161/HYPERTENSIONAHA.107.090290

[3] Feihl F, Liaudet L, Levy BI, Waeber B (2008) Hypertension and microvascular remodelling. Cardiovasc Res 78: 274–285. 18250145 10.1093/cvr/cvn022

[4] Feihl F, Liaudet L, Waeber B (2009) The macrocirculation and microcirculation of hypertension. Curr Hypertens Rep 11: 182–189. 19442327 10.1007/s11906-009-0033-6

[5] Mulvany MJ (2008) Small artery remodelling in hypertension: causes, consequences and therapeutic implications. Med Biol Eng Comput 46: 461–467. 18228071 10.1007/s11517-008-0305-3

[6] Feihl F, Liaudet L, Waeber B, Levy BI (2006) Hypertension: a disease of the microcirculation? Hypertension 48: 1012–1017. 17060505 10.1161/01.HYP.0000249510.20326.72

[7] Folkow B (1982) Physiological aspects of primary hypertension. Physiol Rev 62: 347–504. 6461865 10.1152/physrev.1982.62.2.347

[8] Owens AP (2010) Angiotensin II induces a region-specific hyperplasia of the ascending aorta through regulation of inhibitor of differentiation 3. Circ Res 106: 611–619. 20019328 10.1161/CIRCRESAHA.109.212837 PMC2825288

[9] Owens GK, Schwartz SM (1983) Vascular smooth muscle cell hypertrophy and hyperploidy in the Goldblatt hypertensive rat. Circ Res 53: 491–501. 6627608 10.1161/01.res.53.4.491

[10] Castro MM, Tanus-Santos JE, Gerlach RF (2011) Matrix metalloproteinases: targets for doxycycline to prevent the vascular alterations of hypertension. Pharmacol Res 64: 567–572. 21514386 10.1016/j.phrs.2011.04.002

[11] Castoldi G, Di Gioia CR, Pieruzzi F, D'Orlando C, Van De Greef WM, et al (2003) ANG II increases TIMP-1 expression in rat aortic smooth muscle cells in vivo. Am J Physiol Heart Circ Physiol 284: H635–643. 12388255 10.1152/ajpheart.00986.2001

[12] Raffetto JD, Khalil RA (2008) Matrix metalloproteinases and their inhibitors in vascular remodeling and vascular disease. Biochem Pharmacol 75: 346–359. 17678629 10.1016/j.bcp.2007.07.004 PMC2254136

[13] Wang X, Robbins J (2006) Heart failure and protein quality control. Circ Res 99: 1315–1328. 17158347 10.1161/01.RES.0000252342.61447.a2

[14] Depre C, Powell SR, Wang X (2010) The role of the ubiquitin-proteasome pathway in cardiovascular disease. Cardiovasc Res 85: 251–252. 19892772 10.1093/cvr/cvp362

[15] Li YF, Wang X (2011) The role of the proteasome in heart disease. Biochim Biophys Acta 1809: 141–149. 20840877 10.1016/j.bbagrm.2010.09.001 PMC3021001

[16] Pashevin DA, Tumanovska LV, Dosenko VE, Nagibin VS, Gurianova VL, et al (2011) Antiatherogenic effect of quercetin is mediated by proteasome inhibition in the aorta and circulating leukocytes. Pharmacol Rep 63: 1009–1018. 22001989 10.1016/s1734-1140(11)70617-x

[17] Xie P, Fan Y, Zhang H, Zhang Y, She M, et al (2009) CHIP represses myocardin-induced smooth muscle cell differentiation via ubiquitin-mediated proteasomal degradation. Mol Cell Biol 29: 2398–2408. 19237536 10.1128/MCB.01737-08 PMC2668377

[18] Demasi M, Laurindo FR (2012) Physiological and pathological role of the ubiquitin-proteasome system in the vascular smooth muscle cell. Cardiovasc Res 95: 183–193. 22451513 10.1093/cvr/cvs128

[19] Takaoka M, Ohkita M, Itoh M, Kobayashi Y, Okamoto H, et al (2001) A proteasome inhibitor prevents vascular hypertrophy in deoxycorticosterone acetate-salt hypertensive rats. Clin Exp Pharmacol Physiol 28: 466–468. 11380524

[20] Takaoka M, Okamoto H, Ito M, Nishioka M, Kita S, et al (1998) Antihypertensive effect of a proteasome inhibitor in DOCA-salt hypertensive rats. Life Sci 63: PL65–70. 9698041 10.1016/s0024-3205(98)00276-8

[21] Xu J, Wang S, Wu Y, Song P, Zou MH (2009) Tyrosine nitration of PA700 activates the 26S proteasome to induce endothelial dysfunction in mice with angiotensin II-induced hypertension. Hypertension 54: 625–632. 19597039 10.1161/HYPERTENSIONAHA.109.133736 PMC2910588

[22] Ludwig A, Fechner M, Wilck N, Meiners S, Grimbo N, et al (2009) Potent anti-inflammatory effects of low-dose proteasome inhibition in the vascular system. J Mol Med (Berl) 87: 793–802. 19399470 10.1007/s00109-009-0469-9

[23] Meiners S, Hocher B, Weller A, Laule M, Stangl V, et al (2004) Downregulation of matrix metalloproteinases and collagens and suppression of cardiac fibrosis by inhibition of the proteasome. Hypertension 44: 471–477. 15337735 10.1161/01.HYP.0000142772.71367.65

[24] Papandreou CN, Daliani DD, Nix D, Yang H, Madden T, et al (2004) Phase I trial of the proteasome inhibitor bortezomib in patients with advanced solid tumors with observations in androgen-independent prostate cancer. J Clin Oncol 22: 2108–2121. 15169797 10.1200/JCO.2004.02.106

[25] Kuroki MT, Guzman PA, Fink GD, Osborn JW (2012) Time-dependent changes in autonomic control of splanchnic vascular resistance and heart rate in ANG II-salt hypertension. Am J Physiol Heart Circ Physiol 302: H763–769. 22114134 10.1152/ajpheart.00930.2011 PMC3353774

[26] Simon G, Cserep G, Limas C (1995) Development of structural vascular changes with subpressor angiotensin II administration in rats. Am J Hypertens 8: 67–73. 7734100 10.1016/0895-7061(94)00192-E

[27] Meiners S, Dreger H, Fechner M, Bieler S, Rother W, et al (2008) Suppression of cardiomyocyte hypertrophy by inhibition of the ubiquitin-proteasome system. Hypertension 51: 302–308. 18086945 10.1161/HYPERTENSIONAHA.107.097816

[28] Cavaletti G, Gilardini A, Canta A, Rigamonti L, Rodriguez-Menendez V, et al (2007) Bortezomib-induced peripheral neurotoxicity: a neurophysiological and pathological study in the rat. Exp Neurol 204: 317–325. 17214983 10.1016/j.expneurol.2006.11.010

[29] Semprun-Prieto LC, Sukhanov S, Yoshida T, Rezk BM, Gonzalez-Villalobos RA, et al (2011) Angiotensin II induced catabolic effect and muscle atrophy are redox dependent. Biochem Biophys Res Commun 409: 217–221. 21570954 10.1016/j.bbrc.2011.04.122 PMC3109128

[30] Meiners S, Heyken D, Weller A, Ludwig A, Stangl K, et al (2003) Inhibition of proteasome activity induces concerted expression of proteasome genes and de novo formation of Mammalian proteasomes. J Biol Chem 278: 21517–21525. 12676932 10.1074/jbc.M301032200

[31] Kloss A, Meiners S, Ludwig A, Dahlmann B (2010) Multiple cardiac proteasome subtypes differ in their susceptibility to proteasome inhibitors. Cardiovasc Res 85: 367–375. 19564153 10.1093/cvr/cvp217

[32] Mitchell GF (2008) Effects of central arterial aging on the structure and function of the peripheral vasculature: implications for end-organ damage. J Appl Physiol 105: 1652–1660. 18772322 10.1152/japplphysiol.90549.2008 PMC2584844

[33] Mitchell GF, Parise H, Benjamin EJ, Larson MG, Keyes MJ, et al (2004) Changes in arterial stiffness and wave reflection with advancing age in healthy men and women: the Framingham Heart Study. Hypertension 43: 1239–1245. 15123572 10.1161/01.HYP.0000128420.01881.aa

[34] Henrich H, Hertel R, Assmann R (1978) Structural differences in the mesentery microcirculation between normotensive and spontaneously hypertensive rats. Pflugers Arch 375: 153–159. 567786 10.1007/BF00584238

[35] Korsgaard N, Mulvany MJ (1988) Cellular hypertrophy in mesenteric resistance vessels from renal hypertensive rats. Hypertension 12: 162–167. 3410524 10.1161/01.hyp.12.2.162

[36] Rizzoni D, Porteri E, Guefi D, Piccoli A, Castellano M, et al (2000) Cellular hypertrophy in subcutaneous small arteries of patients with renovascular hypertension. Hypertension 35: 931–935. 10775564 10.1161/01.hyp.35.4.931

[37] Brouwers-Ceiler DL, Nelissen-Vrancken HJ, Smits JF, De Mey JG (1997) The influence of angiotensin II-induced increase in aortic wall mass on compliance in rats in vivo. Cardiovasc Res 33: 478–484. 9074713 10.1016/s0008-6363(96)00213-1

[38] Androulakis E, Tousoulis D, Papageorgiou N, Latsios G, Siasos G, et al (2012) The role of matrix metalloproteinases in essential hypertension. Curr Top Med Chem 12: 1149–1158. 22519445 10.2174/1568026611208011149

[39] Belmadani S, Zerfaoui M, Boulares HA, Palen DI, Matrougui K (2008) Microvessel vascular smooth muscle cells contribute to collagen type I deposition through ERK1/2 MAP kinase, alphavbeta3-integrin, and TGF-beta1 in response to ANG II and high glucose. Am J Physiol Heart Circ Physiol 295: H69–76. 18456735 10.1152/ajpheart.00341.2008 PMC2494762

[40] Deguchi JO, Huang H, Libby P, Aikawa E, Whittaker P, et al (2009) Genetically engineered resistance for MMP collagenases promotes abdominal aortic aneurysm formation in mice infused with angiotensin II. Lab Invest 89: 315–326. 19153555 10.1038/labinvest.2008.167 PMC2932654

[41] Castoldi G, di Gioia CR, Travaglini C, Busca G, Redaelli S, et al (2007) Angiotensin II increases tissue-specific inhibitor of metalloproteinase-2 expression in rat aortic smooth muscle cells in vivo: evidence of a pressure-independent effect. Clin Exp Pharmacol Physiol 34: 205–209. 17250640 10.1111/j.1440-1681.2007.04573.x

[42] Walter A, Etienne-Selloum N, Sarr M, Kane MO, Beretz A, et al (2008) Angiotensin II induces the vascular expression of VEGF and MMP-2 in vivo: preventive effect of red wine polyphenols. J Vasc Res 45: 386–394. 18354258 10.1159/000121408

[43] Flamant M, Placier S, Dubroca C, Esposito B, Lopes I, et al (2007) Role of matrix metalloproteinases in early hypertensive vascular remodeling. Hypertension 50: 212–218. 17515450 10.1161/HYPERTENSIONAHA.107.089631

[44] Watts SW, Rondelli C, Thakali K, Li X, Uhal B, et al (2007) Morphological and biochemical characterization of remodeling in aorta and vena cava of DOCA-salt hypertensive rats. Am J Physiol Heart Circ Physiol 292: H2438–2448. 17237246 10.1152/ajpheart.00900.2006

[45] Derosa G, D'Angelo A, Ciccarelli L, Piccinni MN, Pricolo F, et al (2006) Matrix metalloproteinase-2, -9, and tissue inhibitor of metalloproteinase-1 in patients with hypertension. Endothelium 13: 227–231. 16840178 10.1080/10623320600780942

[46] Marfella R, D'Amico M, Esposito K, Baldi A, Di Filippo C, et al (2006) The ubiquitin-proteasome system and inflammatory activity in diabetic atherosclerotic plaques: effects of rosiglitazone treatment. Diabetes 55: 622–632. 16505224 10.2337/diabetes.55.03.06.db05-0832

[47] Griendling KK, Ushio-Fukai M, Lassegue B, Alexander RW (1997) Angiotensin II signaling in vascular smooth muscle. New concepts. Hypertension 29: 366–373. 9039129 10.1161/01.hyp.29.1.366

[48] Dreger H, Westphal K, Weller A, Baumann G, Stangl V, et al (2009) Nrf2-dependent upregulation of antioxidative enzymes: a novel pathway for proteasome inhibitor-mediated cardioprotection. Cardiovasc Res 83: 354–361. 19351736 10.1093/cvr/cvp107

[49] Leak RK, Zigmond MJ, Liou AK (2008) Adaptation to chronic MG132 reduces oxidative toxicity by a CuZnSOD-dependent mechanism. J Neurochem 106: 860–874. 18466318 10.1111/j.1471-4159.2008.05459.x PMC2901869

[50] Kim SY, Lee JH, Huh JW, Kim HJ, Park MK, et al (2012) Bortezomib alleviates experimental pulmonary arterial hypertension. Am J Respir Cell Mol Biol 47: 698–708. 22842494 10.1165/rcmb.2011-0331OC

[51] Stangl V, Lorenz M, Meiners S, Ludwig A, Bartsch C, et al (2004) Long-term up-regulation of eNOS and improvement of endothelial function by inhibition of the ubiquitin-proteasome pathway. FASEB J 18: 272–279. 14769821 10.1096/fj.03-0054com

[52] Lorenz M, Wilck N, Meiners S, Ludwig A, Baumann G, et al (2009) Proteasome inhibition prevents experimentally-induced endothelial dysfunction. Life Sci 84: 929–934. 19409913 10.1016/j.lfs.2009.04.016

[53] Kim W, Chapkin RS, Barhoumi R, Ma DW (2009) A novel role for nutrition in the alteration of functional microdomains on the cell surface. Methods Mol Biol 579: 261–270. 19763480 10.1007/978-1-60761-322-0_13 PMC2821694

[54] Harrison DG, Marvar PJ, Titze JM (2012) Vascular inflammatory cells in hypertension. Front Physiol 3: 128. 22586409 10.3389/fphys.2012.00128 PMC3345946

[55] Kim SC, Rho MC, Lee HS, Kim YK, Kim K (2003) Caspase-3-dependent apoptosis in vascular smooth muscle cell by proteasome inhibition. J Cardiovasc Pharmacol 42: 554–560. 14508242 10.1097/00005344-200310000-00014

[56] Meiners S, Ludwig A, Stangl V, Stangl K (2008) Proteasome inhibitors: poisons and remedies. Med Res Rev 28: 309–327. 17880010 10.1002/med.20111

[57] Rizzoni D, Muiesan ML, Porteri E, De Ciuceis C, Boari GE, et al (2009) Vascular remodeling, macro- and microvessels: therapeutic implications. Blood Press 18: 242–246. 19919394 10.3109/08037050903254923

